# Synthesis and Cytotoxicity Assessment of Citrate-Coated Calcium and Manganese Ferrite Nanoparticles for Magnetic Hyperthermia

**DOI:** 10.3390/pharmaceutics14122694

**Published:** 2022-12-01

**Authors:** Raquel G. D. Andrade, Débora Ferreira, Sérgio R. S. Veloso, Cátia Santos-Pereira, Elisabete M. S. Castanheira, Manuela Côrte-Real, Ligia R. Rodrigues

**Affiliations:** 1 Physics Centre of Minho and Porto Universities (CF-UM-UP), University of Minho, Campus de Gualtar, 4710-057 Braga, Portugal; 2 LaPMET (Laboratory of Physics for Materials and Emergent Technologies), Associate Laboratory, 4710-057 Braga, Portugal; 3 CEB—Centre of Biological Engineering, University of Minho, Campus de Gualtar, 4710-057 Braga, Portugal; 4 LABBELS—Associate Laboratory, 4710-057 Braga, Portugal; 5 Centre of Molecular and Environmental Biology (CBMA), Department of Biology, University of Minho, 4710-057 Braga, Portugal

**Keywords:** magnetic hyperthermia, magnetic nanoparticles, calcium-doped manganese ferrite, cellular internalization, cancer treatment

## Abstract

Calcium-doped manganese ferrite nanoparticles (NPs) are gaining special interest in the biomedical field due to their lower cytotoxicity compared with other ferrites, and the fact that they have improved magnetic properties. Magnetic hyperthermia (MH) is an alternative cancer treatment, in which magnetic nanoparticles promote local heating that can lead to the apoptosis of cancer cells. In this work, manganese/calcium ferrite NPs coated with citrate (Ca*_x_*Mn_1−*x*_Fe_2_O_4_ (*x* = 0, 0.2, 1), were synthesized by the sol-gel method, followed by calcination, and then characterized regarding their crystalline structure (by X-ray diffraction, XRD), size and shape (by Transmission Electron Microscopy, TEM), hydrodynamic size and zeta potential (by Dynamic Light Scattering, DLS), and heating efficiency (measuring the Specific Absorption Rate, SAR, and Intrinsic Loss Power, ILP) under an alternating magnetic field. The obtained NPs showed a particle size within the range of 10 nm to 20 nm (by TEM) with a spherical or cubic shape. Ca_0.2_Mn_0.8_Fe_2_O_4_ NPs exhibited the highest SAR value of 36.3 W/g at the lowest field frequency tested, and achieved a temperature variation of ~7 °C in 120 s, meaning that these NPs are suitable magnetic hyperthermia agents. In vitro cellular internalization and cytotoxicity experiments, performed using the human cell line HEK 293T, confirmed cytocompatibility over 0–250 µg/mL range and successful internalization after 24 h. Based on these studies, our data suggest that these manganese-calcium ferrite NPs have potential for MH application and further use in in vivo systems.

## 1. Introduction

Cancer is still one of the major burdens of contemporary society. Despite all the efforts to control this disease, current treatments fail to provide a long-term survival rate while improving patient compliance, mostly due to tumors’ heterogeneity and acquired resistance [1]. Nanomedicine is a field of nanotechnology that provides versatile platforms for a precise and efficient therapy. Nanoparticles (NPs) are small-sized (1–1000 nm) systems with unique structural and physicochemical properties that can be used in cancer treatment and diagnosis (nanotheranostics) as multi-modal agents [2]. In particular, superparamagnetic iron-oxide NPs have been widely exploited for their important features in this field, namely stability, biocompatibility, high magnetization, improved contrast enhancement for bioimaging, surface coating to avoid agglomeration, and conjugation with different targeting moieties and drugs. These properties enable a magnetically-driven and site-specific drug delivery [3,4]. Another important feature used in cancer treatment is the ability to generate heat, thus allowing the performance of magnetic hyperthermia (MH). 

MH is a heat-triggered cancer treatment in which magnetic nanoparticles generate heat under an alternating magnetic field (AMF), with the objective of increasing the local temperature (up to 42–45 °C) and promoting the apoptosis of cancer cells or making them more sensitive to other treatments [5]. MH efficiency is determined through the value of SAR, which depends on the NPs’ size, shape, composition of ferrites, concentration, and applied magnetic field. High values of SAR allow the decrease of the necessary dosage and time of treatment, but without neglecting the particle size, saturation magnetization and the time, frequency, and amplitude of the applied magnetic field needed to achieve a therapeutic temperature [6]. Therefore, it is essential to accomplish a balance between the magnetic properties and biocompatibility of NPs. In order to find this balance, several ferrite compositions are being explored by combining different metal cations and varying their concentration. Among the used ferrites for MH, manganese ferrites (MnFe_2_O_4_) exhibit low inherent toxicity, chemical stability, ease of synthesis and excellent magnetic properties, with reported suitability as an enhanced magnetic resonance imaging (MRI) contrast agent [7,8]. The concern about the cytotoxicity of these ferrites has led to the substitution of heavy metals by other cations, such as calcium, resulting in mixed ferrites with higher biocompatibility and preserved magnetic properties [9,10]. Besides that, a suitable surface modification is necessary to avoid NP aggregation and improve colloidal stability.

In this work, citrate-coated calcium-substituted manganese ferrite NPs (Ca*_x_*Mn_1−*x*_Fe_2_O_4_, *x* = 0, 0.2, 1) were synthesized by the sol-gel method and characterized regarding their crystalline structure (by XRD), shape and size (by TEM), and heating efficiency (SAR and ILP). Cytotoxicity and internalization experiments were performed in the human embryonic kidney HEK 293T cell line to assess if the prepared NPs are suitable for future application in clinical magnetic hyperthermia.

## 2. Materials and Methods

### 2.1. Preparation of Citrate-Coated Calcium-Doped Manganese Ferrite NPs

The mixed Ca*_x_*Mn_1-*x*_Fe_2_O_4_ NPs were prepared by the sol-gel method. Firstly, 10 mL containing stoichiometric amounts of mixed Fe(NO_3_)_3_·9H_2_O, Mn(NO_3_)_2_·4H_2_O and Ca(NO_3_)_2_·4H_2_O were added to 10 mL of a 0.6 M citric acid solution, and the mixture was stirred for 2 h at 80 °C. Then, the temperature was raised to 90 °C and the mixture was left to dry, without stirring, until formation of the xerogel. Finally, the xerogel was left drying at 120 °C for 2 h, and overnight at 90 °C. The obtained NPs were subjected to calcination at 500 °C in the case of Ca-doped manganese ferrites and at 300 °C in the case of MnFe_2_O_4_. To functionalize the NPs with citrate, 50 mg of NPs were dispersed in 10 mL of trisodium citrate 0.3 M at pH = 7, and sonicated for 30 min. Then, the solution was stirred at 80 °C for 2 h, washed with water and acetone, and left dry at 100 °C overnight.

### 2.2. Preparation of Carboxyfluorescein-Functionalized Ca_0.2_Mn_0.8_Fe_2_O_4_ NPs

To evaluate the cellular internalization of NPs, citrate-functionalized Ca_0.2_Mn_0.8_Fe_2_O_4_ NPs were coupled to 5(6)-carboxyfluorescein (CF) (Sigma-Aldrich). Firstly, CF (0.01 mmol/mg) and 2 equivalents of *N*,*N*,*N*’,*N*’-Tetramethyl-O-(1*H*-benzotriazol-1-yl)uronium hexafluorophosphate (HBTU) (Sigma-Aldrich, St. Louis, MA, USA) were mixed in dimethylformamide (DMF), and a solution of 5 mg of NPs in 15 mL of DMF was added to a final solution volume of 20 mL. Then, triethylamine was added (2.2 equivalents) and the reaction was left stirring at room temperature (RT) overnight. After that, NPs were washed with DMF, ethanol, and ultrapure water and dried overnight at RT [11].

### 2.3. Nanoparticle Characterization

#### 2.3.1. X-ray Diffraction (XRD)

The phase identification and crystalline structure analysis of the NPs were determined by XRD using a PAN’alytical X’Pert PRO diffractometer (Malvern Panalytical Ltd., Malvern, UK) with a CuK_α_ radiation (λ = 0.154060 nm) in a Bragg-Brentano configuration, at the Electron Microscopy Unit, University of Trás-os-Montes and Alto Douro (UTAD), Vila Real, Portugal. 

#### 2.3.2. Transmission Electron Microscopy (TEM)

TEM images were obtained using a high contrast JEOL JEM-1010, operating at 100 kV (CACTI, Vigo, Spain). A small portion of the sample was placed onto a TEM 400 mesh copper grid with Formvar/Carbon (ref. S162-4 from Agar Scientific), held by tweezers, and the excess solution was cleaned. TEM images were analyzed using ImageJ software (Version 1.51q, National Institutes of Health, Bethesda, MD, USA), by manually selecting the NPs to estimate the particle size.

#### 2.3.3. Dynamic Light Scattering (DLS)

The average hydrodynamic size and zeta potential of the prepared NPs were measured in phosphate buffer pH 7.4 at 0.01 M and obtained with a DLS equipment Litesizer™ 500 from Anton Paar (Anton Paar GmbH, Graz, Austria), using a semiconductor laser diode of 40 mW and λ = 658 nm with controlled temperature of 25 °C. The data collected are from two or three different batches (0.01 mg/mL prepared from an initial solution of 2 mg/mL). All the samples were filtered and sonicated before the measurements (3 independent measurements for each sample).

### 2.4. Hyperthermia Measurements

The heating performance of the prepared NPs was evaluated through magneto-caloric measurements carried out using hyperthermia system magneTherm (nanoTherics, Warrington, UK), working at f ≈ 162; 271; 383 and 617 kHz, and at a magnetic field H = 17; 16; or 10 mT. For all experiments, the initial temperature was stabilized before starting the measurement. Next, the AC magnetic field was applied for 10 min and the temperature variation was recorded using a thermocouple.

### 2.5. Cell Culture

The human embryonic kidney HEK 293T (ATCC CRL-3216) cell line was cultured in Dulbecco’s minimal essential medium (DMEM) (Biochrom), supplemented with 10% heat-inactivated fetal bovine serum (FBS) (Biochrom) and 1% penicillin-streptomycin (Biochrom). Cells were grown in polystyrene tissue culture flasks in a humidified atmosphere of 5% CO_2_ at 37 °C, and subcultured using 0.25% Trypsin-EDTA solution (Sigma Aldrich, St. Louis, MO, USA).

### 2.6. In Vitro Cytotoxicity Assay

The effect of the citrate-functionalized NPs on cell viability was assessed using the MTT assay. For this purpose, 1 × 10^4^ HEK 293T cells were plated on 96-well culture plates and incubated overnight. Then, cells were incubated with each type of NPs at concentrations of 100, 150, 200, and 250 µg/mL for 24 h and 48 h. After incubation, the medium was removed and 0.5 mg/mL of 3-(4,5-Dimethylthiazol-2-yl)-2,5-Diphenyltetrazolium Bromide (MTT) (Sigma Aldrich, St. Louis, MO, USA) solution was added to each well and incubated for 2 h at 37 °C. The blue formazan crystals formed by viable cells were dissolved in dimethylsulfoxide (DMSO) (Thermo Fisher Scientific, Waltham, MA, USA), and their optical density was measured at a wavelength of 570 nm in a microplate reader (Cytation 3, BioTek, Winooski, VT, USA).

### 2.7. Intracellular Uptake by Fluorescence Microscopy Analysis

To visualize the cellular uptake of NPs, citrate-coated Ca_0.2_Mn_0.8_Fe_2_O_4_ NPs were functionalized with carboxyfluorescein, as described above. HEK 293T cells were seeded on coverslips in a 6-well plate (2 × 10^5^ cell/well) and incubated with NPs at 200 µg/mL for 24 h. After incubation, cells were fixed with 4% paraformaldehyde (PFA) (Sigma Aldrich, St. Louis, MO, USA) for 40 min at RT and then stained with 4′,6-diamidino-2-phenylindole (DAPI) (Biotium, San Francisco, CA, USA) for 15 min at RT. After that, cells were washed with phosphate buffered saline (PBS) pH = 7.4, mounted on a glass slide, and visualized using a 40× objective in a fluorescence microscope (OLYMPUS BX51) incorporated with high-sensitivity camera Olympus DP71. Images were analyzed by ImageJ software.

## 3. Results and Discussion

### 3.1. Nanoparticles Characterization by XRD and TEM

**Figure 1:**
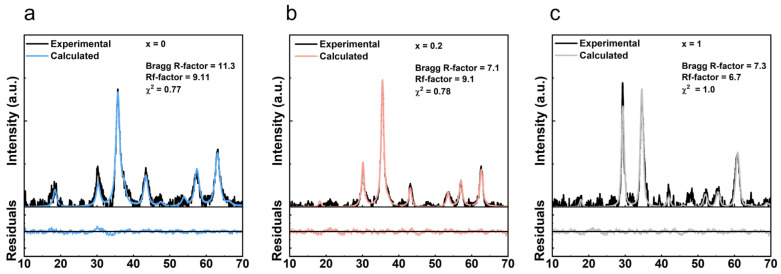
XRD diffraction patterns for Ca*_x_*Mn_1-*x*_Fe_2_O_4_ ferrites: (**a**) *x* = 0; (**b**) *x* = 0.2; (**c**) *x* = 1, and fitted patterns obtained through Rietveld refinement.

The diffraction patterns of the synthesized NPs were assessed to determine the phase composition and size of the NPs. The crystalline phase was assessed through Rietveld refinement using the FullProf software suite (Figure 1). The diffractograms were fitted to a cubic spinel phase (space group Fd3m, adapted from magnetite CIF 2300618), and using, as background, a set of 23 equally spaced points with adjustable intensities joined by linear regression lines. In general, the profiles displayed the well-defined peaks characteristic of a crystalline structure, and, as suggested by the good fitting from the Rietveld refinement, the Bragg’s reflections characteristic of the Fd3m space group point out the presence of manganese/calcium ferrites with a cubic structure [12,13,14,15,16,17]. Notably, the presently employed method did not result in the formation of significant amounts of hematite as those reported in other sol-gel methods [14]. The average crystallite sizes were determined from the diffraction peak, with higher intensity corresponding to the reflection plane (3 1 1), using the Scherrer’s equation [18]:

(1) $$d=\frac{k\lambda }{\beta cosϴ}$$

where *k* is the correction factor (*k* = 0.89), λ is the wavelength of CuK_α_ which is 1.5406 Å, *β* is the FWHM of the (3 1 1) peak, and *ϴ* is the Bragg’s angle [12,19].

Table 1 shows the XRD parameters for MnFe_2_O_4_ (*x* = 0) and Ca-doped Mn ferrites (*x* = 0.2 and *x* = 1). The crystallite size increased with the amount of calcium until *x* = 0.2, and then decreased when *x* = 1. An increase of particle size and crystallite size with the incremental calcium content for calcium doped nickel ferrite NPs was described elsewhere [20]. The authors associated the increasing crystallite size with the occupation of the tetrahedral A-sites by the Ca^2+^ ions. However, the non-linear dependence suggests that other parameters related to the synthesis method might affect the crystallite size at higher concentrations of Ca^2+^, in addition to the differences arising from changes in the cation distribution.

**Figure 2:**
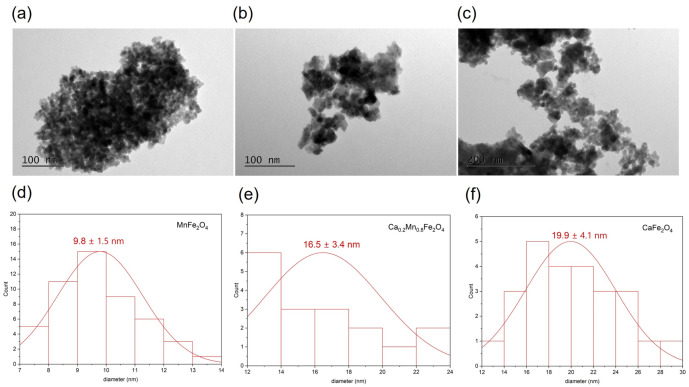
TEM images of MnFe_2_O_4_ (**a**), Ca_0.2_Mn_0.8_Fe_2_O_4_ (**b**) and CaFe_2_O_4_ (**c**) NPs and corresponding histograms of size distribution (**d**–**f**).

Figure 2 shows the TEM images of the prepared NPs without citrate. The images demonstrate that the morphology of the NPs changed with the addition of calcium, from a general spherical shape, observed for MnFe_2_O_4_ NPs, to a mixture of spherical and cubic shapes, seen in the NPs containing calcium. A Gaussian distribution was fitted to the experimental data and revealed an increase in the particle size with the increase in substitution of Ca^2+,^ from 10 nm to 20 nm. A size of 11 nm was already reported for Ca_0.2_Mn_0.8_Fe_2_O_4_ NPs treated at 400 °C. The NPs obtained in this work were calcinated at 500 °C, which can lead to the formation of more complete crystalline phases [21,22]. The trend in size change obtained from TEM reveals an increase in particle size with the percentage of calcium.

### 3.2. Nanoparticles Characterization by DLS

**Figure 3:**
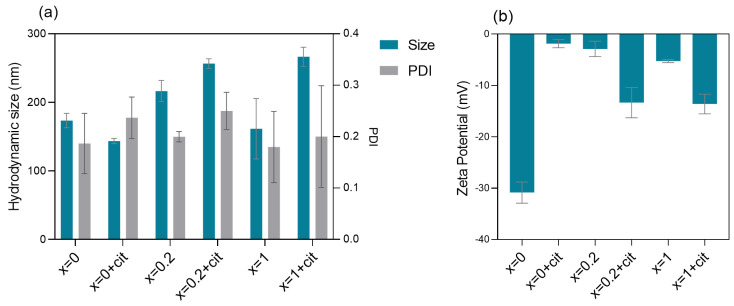
Hydrodynamic size, PDI value (**a**) and zeta potential (**b**) of the bare and citrate-functionalized Ca_*x*_Mn_1-*x*_Fe_2_O_4_ NPs at 0.01 mg/mL in phosphate buffer pH 7.4. Each value represents the mean ± SD (SD: standard deviation) of two or three independent measurements. Abbreviations: *x* = 0 + cit, *x* = 0.2 + cit and *x* = 1 + cit are citrate−functionalized MnFe_2_O_4_, Ca_0.2_Mn_0.8_Fe_2_O_4_ and CaFe_2_O_4_ nanoparticles, respectively.

Considering the influence of physicochemical parameters such as size, surface charge, and coating in the behavior of NPs in biological environments and interaction with cells, dynamic light scattering and electrophoretic light scattering were performed in a phosphate buffer 0.01 M pH = 7.4 to obtain the hydrodynamic size, polydispersity index (PDI), and zeta potential values (Figure 3).

Figure 3a shows the dependence of hydrodynamic size and PDI on the presence of citrate and quantity of calcium. The hydrodynamic size, which refers to the particle size associated with the hydration layer around them, was ~200 nm for bare NPs and in the 200–300 nm range for citrate-functionalized NPs, pointing to some aggregation of the particles in the buffer used.

Citrate-functionalized Ca_0.2_Mn_0.8_Fe_2_O_4_ and CaFe_2_O_4_ NPs show the highest sizes due to the calcium ions’ substitution and the presence of citrate molecules that promote the increase of the hydration layer, thus confirming the functionalization. PDI indicates the degree of dispersion of a particle in a colloidal suspension, and therefore reflects the degree of agglomeration. The obtained PDI values were in the 0.20–0.30 range, which was reported to be an acceptable value for drug delivery applications [23], and meaning that the samples are not very polydisperse. Zeta potential is a measure of the stability and surface coating of NPs, and is indicative of the surface charge in the colloidal suspension. A stable colloidal solution has zeta potential values lower than −30 mV and higher than +30 mV [24]. In general, the zeta potential results (Figure 3b) show that all the NPs present negative charge. Zeta potential values are lower for citrate-functionalized Ca-Mn ferrites compared with the ones without citrate, due to the carboxylic terminal groups of citrate molecules [25]. However, the values are less negative than −30 mV, which could indicate an agglomeration tendency in these particles (as inferred from the hydrodynamic size values).

### 3.3. Magnetic Hyperthermia (MH) Measurements 

The heating efficiencies of the synthesized citrate-coated NPs dispersed in ultrapure water at a concentration of 5 mg/mL were evaluated under an AMF at four different conditions of field strengths and frequencies, below the biological acceptance limit of H_AC_ × f= 2 × 10^9^ A m^−1^ s^−1^ [26]: (a) 7.98 kA/m and 616 kHz, (b) 12.76 kA/m and 382.6 kHz, (c) 13.56 kA/m and 270.6 kHz and (d) 13.56 kA/m and 161.6 kHz.

The heating efficiency can be quantified through the value of SAR, which can be defined as the heat generated by a magnetic NP per unit gram of magnetic material per unit time, and can be calculated through Equation (2):

(2) $$\mathrm{SAR}=\frac{C}{m_{NP}}\frac{\Delta T}{\Delta t}$$

where *C* is the specific heat capacity of suspension, *m_NP_* is the mass of nanoparticles per unit volume and ∆*T*/∆*t* is the initial slope of the temperature (*T*) vs. time (*t*) curve [27]. Despite SAR values usually being reported to characterize the heating efficiency of NPs, its comparison is limited by its dependence on the frequency and amplitude of the AMF used in the experiments. Thus, the SAR values of superparamagnetic NPs can be normalized to the value of ILP through the following Equation (3):

(3) $$\mathrm{ILP}=\frac{\mathrm{SAR}}{H^{2}f}$$

where $H^{2}f$ is the product of the field strength (kA/m) and frequency (kHz) [28].

**Figure 4:**
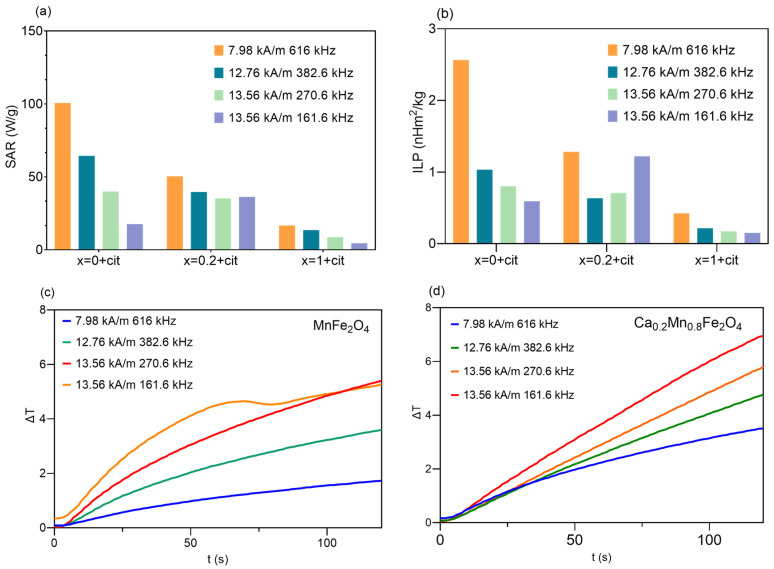
Values of SAR (**a**), ILP (**b**) of citrate-functionalized NPs and variation of temperature (ΔT) over time (t) of MnFe_2_O_4_ (**c**), and Ca_0.2_Mn_0.8_Fe_2_O_4_ NPs (**d**), under an AMF at different conditions below the biological acceptance limit (H_AC_ × f = 2 × 10^9^ A m^−1^ s^−1^). Abbreviations: *x* = 0 + cit, *x* = 0.2 + cit and *x* = 1 + cit are citrate-functionalized MnFe_2_O_4_, Ca_0.2_Mn_0.8_Fe_2_O_4_, and CaFe_2_O_4_ nanoparticles, respectively.

Figure 4a,b shows SAR and ILP values for each NP at the measured conditions. MnFe_2_O_4_ NPs exhibited the highest SAR (100.7 W/g) and ILP (2.6 nH·m^2^/kg) values at 7.98 kA/m and 616 kHz, which is in agreement with already reported values for MnFe_2_O_4_ or mixed MnFe_2_O_4_ NPs [29,30,31,32]. In general, for the same conditions, the increase of calcium content led to a decrease in the heating efficiency, except at 13.56 kA/m and 161.6 kHz, at which SAR and ILP values of Ca_0.2_Mn_0.8_Fe_2_O_4_ NPs were the highest, achieving 36.3 W/g and 1.22 nH·m^2^/kg, respectively. This means that these NPs show significant heating capability at the lowest field frequency studied.

Figure 4c,d shows the plots of the variation of temperature versus time of the citrate-functionalized MnFe_2_O_4_ and Ca_0.2_Mn_0.8_Fe_2_O_4_ NPs. An increase of the heating rate is observable as the amplitude of the field increases, so the amplitude of 13.56 kA/m will be used for comparison. At this amplitude and frequencies of 270.6 kHz and 161.6 kHz, MnFe_2_O_4_ NPs reached a temperature variation of ~5 °C in 120 s, while the presence of 20% calcium led to a better heating performance of the ferrite, achieving a variation of ~6 °C and ~7 °C at 270.6 kHz and 161.6 kHz, respectively. Considering that the viability of cancer cells can be affected by mild hyperthermia (~42 °C), and that the body temperature is around 37 °C, these NPs were capable of achieving the mild hyperthermia-required temperature and acting as hyperthermia agents, which could be controlled through an intermittent time-set technique [33].

Despite the very promising results herein presented, the suitability of these NPs as cancer cells death inducers must be demonstrated through further in vitro hyperthermia studies. It has been described that the magnetic hyperthermia heating profile in cells and in vivo is smaller than the one achieved for the pure nanoparticles in solution [34]. This could be a result of several factors, such as the formation of a protein corona, aggregation of the nanoparticles in cells, and additional impairment of the Brownian relaxation contribution to heat generation. However, despite the smaller heat generation in the biological environment, the nanoparticles have been described to remain effective in inducing cell death [34]. For example, Gupta et al. [35] reported a reduction of ~60% in cell viability of the human glioma U87-MG cell line incubated with Mn-doped ferrite NPs under an AMF at 168 Oe (13.4 kA/m) and 405 kHz. Furthermore, the researchers related the cell death with the visualized distortion of the cellular cytoskeleton, indicative of the heat-induced molecular pathways that lead to an apoptotic cell death [36,37,38].

### 3.4. Cytotoxicity of Citrate-Functionalized Ca-Mn Ferrites on the HEK 293T Cell Line 

**Figure 5:**
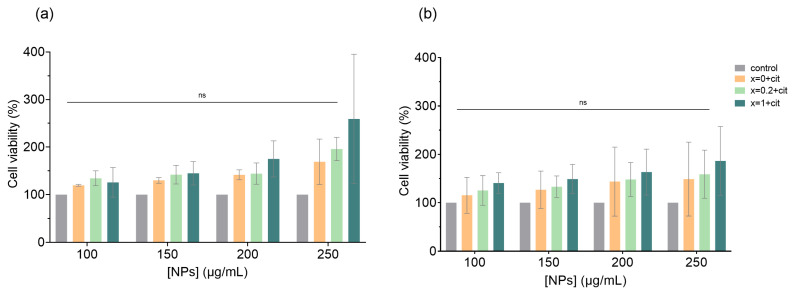
In vitro cytotoxicity in HEK 293T cells of citrate-functionalized Ca-Mn ferrite NPs. Cell viability was assessed by MTT assay in HEK 293T cells incubated with four different concentrations of NPs (100, 150, 200, 250 µg/mL) for 24 h (**a**) and 48 h (**b**). Untreated cells at 24 h and 48 h were used as a negative control (100% viable cells). Data are expressed as the mean of three independent experiments (*n* = 3). Two-way ANOVA assessed by Tukey’s multiple comparison test indicates no statistically significant differences between the groups tested, denoted as ns *p* > 0.05. Abbreviations: *x* = 0 + cit, *x* = 0.2 + cit and *x* = 1 + cit are citrate-functionalized MnFe_2_O_4_, Ca_0.2_Mn_0.8_Fe_2_O_4_ and CaFe_2_O_4_ nanoparticles, respectively.

The in vitro cytotoxicity of citrate-functionalized Ca*_x_*Mn_1-*x*_Fe_2_O_4_ NPs against the proof-of-concept cell line HEK 293T was assessed by the MTT assay. Figure 5 shows the dose dependent cytotoxicity of the NPs at 24 h (a) and 48 h (b). Untreated cells were used as negative control and normalized to 100% cell viability. The results show that for all concentrations tested, cell viability is above 100%. Despite the lack of statistical significance when comparing the different NP’s concentration effect on the cells, it was possible to visualize cell growth after 24 h and 48 h. A cell viability above 90% was also reported for CaFe_2_O_4_ NPs at concentrations below 250 µg/mL on T cell lines [39] for PEG-coated Mn-doped ferrite NPs at concentrations till 750 µg/mL on murine L929 cells [35], and also for Ca-doped MgFe_2_O_4_ NPs up to a concentration of 1000 µg/mL on BLO-11 and NIH-3T3 cells [32]. In this cell line, the replacement of Mn ions for Ca ions did not led to a higher cytocompatibility compared with MnFe_2_O_4_ NPs, so the choice of the better formulation for hyperthermia application will depend on the heating efficiency, magnetic properties and colloidal stability.

### 3.5. Intracellular Uptake of Calcium-Doped Manganese Ferrite NPs

**Figure 6:**
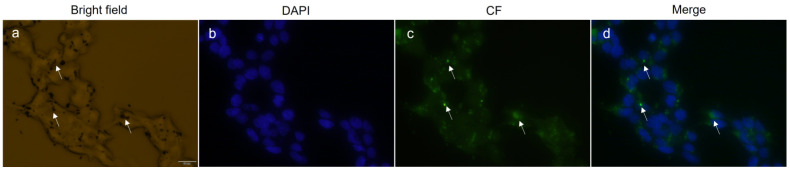
Bright field image of HEK 293T cells containing Ca_0.2_Mn_0.8_Fe_2_O_4_ NPs (**a**), cellular nucleus stained with DAPI (blue) (**b**), NPs coupled with CF (green) (**c**), and overlay of DAPI fluorescence and fluorescence of CF coupled to the NPs (**d**). The white arrows indicate agglomerates of NPs inside the cell. Images were obtained by fluorescence microscopy using a 40× objective. The scale bar corresponds to 20 µm and all images have the same scale.

The intracellular uptake of Ca_0.2_Mn_0.8_Fe_2_O_4_ NPs was investigated on HEK 293T cells by fluorescence microscopy (Figure 6). For that purpose, the cellular nucleus was stained with DAPI and the nanoparticles NPs were coupled with carboxyfluorescein and incubated with cells for 24 h. The bright field image reveals that the NPs, shown as dark spots, entered the cell and formed large aggregates in the cytoplasm that are likely associated with the formation of endosomes filled with NPs, as reported previously [40,41]. Another study reported that cobalt ferrite NPs are internalized via micropinocytosis and clathrin-mediated endocytosis [42], suggesting the same internalization process for the NPs used in this assay. The fluorescence images show the NPs as brighter green spots that match with the location of the dark spots seen in the bright field. The merge of the nucleus and NPs’ fluorescence reveals that after 24 h, some NPs are placed in the proximity of the nucleus. These results led us to conclude that the NPs enter the cell in a nonspecific way, as already reported for other ferrites [43,44,45], and do not promote any cytotoxicity in HEK 293T cells after 24 h, supporting further optimizations in calcium-doped manganese ferrite NPs to develop an efficient cancer therapy agent that may be further exploited for drug delivery.

## 4. Conclusions

The search for more efficient magnetic NPs for cancer theranostic applications is underway, but the concern about their biocompatibility limits the use of some formulations. In this work, calcium/manganese ferrite NPs coated with citrate were synthesized by the sol-gel method in an attempt to obtain mixed ferrites with therapeutic value. TEM measurements show particles’ sizes from 10 nm to 20 nm. The applicability of the prepared NPs for magnetic hyperthermia was demonstrated through the determination of heating efficiencies. Citrate-functionalized MnFe_2_O_4_ and Ca_0.2_Mn_0.8_Fe_2_O_4_ NPs reached a temperature variation of ~5 °C and ~7 °C, respectively, in 120 s at a field amplitude of 13.56 kA/m and the lowest frequency studied of 161.6 kHz. The cytotoxicity assay revealed that these NPs are non-toxic, and fluorescence microscopy experiments showed their internalization after 24 h by HEK 293T cells. Altogether, these results led to the conclusion that the increment of calcium can enhance the hyperthermia ability of these NPs without compromising their cytocompatibility or cellular internalization. Further experiments that aim for optimization related to colloidal stability and surface specificity of these NPs should be performed to create an efficient platform not only for magnetic hyperthermia, but also for drug delivery in cancer cells.

## Figures and Tables

**Table 1:** XRD parameters for Ca*_x_*Mn_1-*x*_Fe_2_O_4_ ferrites, where *x* = 0, *x* = 0.2 and *x* = 1.

Composition (*x*)	2ϴ	FWHM	Crystallite Size (nm)
*x* = 0	35.71	1.27919	6.45
*x* = 0.2	35.48	0.85712	9.62
*x* = 1	34.55	0.97936	8.40

## Data Availability

Not applicable.
